# BOLD Long-Range Temporal Correlations Reflect Changes in Language and Depression Across Intensive Aphasia Therapy

**DOI:** 10.1161/STROKEAHA.124.050064

**Published:** 2025-09-10

**Authors:** Anna-Thekla P. Jäger, Christopher J. Steele, Felix R. Dreyer, Milena R. Osterloh, Anna Sadlon, Vadim Nikulin, Bettina Mohr, Friedemann Pulvermüller

**Affiliations:** Charité Universitätsmedizin, Charité, Berlin, Germany (A.-T.P.J.).; Brain Language Laboratory, Department of Philosophy and Humanities, Freie Universität Berlin, Germany (A.-T.P.J., M.R.O., A.S., F.P.).; Department of Neurology, Max Planck Institute for Human Cognitive and Brain Sciences, Leipzig, Germany (A.-T.P.J., C.J.S., V.N.).; Department of Psychology, School of Health, Concordia University, Montreal, QC, Canada (C.J.S.).; Medizinische Fakultät OWL, Universität Bielefeld, Germany (F.R.D.).; Einstein Center for Neurosciences, Berlin, Germany (M.R.O., F.P.).; ZeNIS-Center for Neuropsychology and Intensive Language Therapy, Berlin, Germany (B.M.).; Cluster of Excellence Matters of Activity, Image Space Material, Humboldt Universität zu Berlin, Germany (F.P.).; Berlin School of Mind and Brain, Humboldt Universität zu Berlin, Germany (F.P.).

**Keywords:** aphasia, depression, fractals, language, magnetic resonance imaging, neuronal plasticity, stroke

## Abstract

**BACKGROUND:**

Intensive language-action therapy treats language deficits and depressive symptoms in chronic poststroke aphasia, yet the underlying neural mechanisms remain underexplored. Long-range temporal correlations (LRTC) in blood oxygenation level–dependent signals indicate persistence in brain activity patterns and may relate to learning and levels of depression. This observational study investigates blood oxygenation level–dependent LRTC changes alongside therapy-induced language and mood improvements in perisylvian and domain-general brain areas.

**METHODS:**

Sixteen patients with chronic poststroke aphasia underwent functional magnetic resonance imaging before and after 2 to 4 weeks of intensive language-action therapy. Therapy took place at Freie Universität Berlin (2014–2020). Language functions and depression were assessed using the Aachen Aphasia Test, the Beck Depression Inventory, and the Montgomery-Åsberg Depression Rating Scale. We implemented a passive reading functional magnetic resonance imaging paradigm and analyzed data using detrended fluctuation analysis to assess LRTC. A 2×2×2 (time, hemisphere, and region of interest) repeated measures ANCOVA (covariates: age, lesion size, time poststroke, and therapy intensity) was conducted in frontoparietal/temporal perisylvian areas across hemispheres before/after therapy. Correlation analyses explored links between changes in behavior and LRTC in focal perisylvian areas and across the whole brain.

**RESULTS:**

Younger patients (relative to the continuous age range of our sample) showed reductions in LRTC across therapy, whereas relatively older patients tended toward increases. We found that changes in LRTC correlated with changes in language performance in right hemisphere perisylvian regions and bilateral domain-general and memory areas (eg, hippocampus, thalamus, supplementary motor area, and putamen). Similarly, changes in depressive symptoms correlated with LRTC changes in right hemisphere perisylvian regions.

**CONCLUSIONS:**

LRTC changes across therapy reflect changes in both language performance and depression in chronic poststroke aphasia. Predominantly right perisylvian and domain-general regions seem critical for neuroplasticity in language rehabilitation. In addition, the observed role of right perisylvian regions in mood regulation highlights the interconnection of cognitive recovery and emotional well-being. LRTC may represent a valuable biomarker for tracking therapy-related neuroplasticity.

About 40% of patients with stroke experience aphasia, a language impairment resulting primarily from damage to left-hemispheric frontal and temporoparietal brain regions.^1,2^ In many cases, aphasia co-occurs with neuropsychiatric conditions, particularly depressive symptoms, suggesting a close link between emotional well-being and language recovery in patients with aphasia (PWA).^3–6^ Importantly, therapy-related improvements of language functions can be achieved in PWA, even at the chronic stage.^7–9^ Investigating recovery during the chronic stage of stroke is particularly advantageous for measuring language reorganization due to the diminished likelihood of spontaneous recovery at this stage compared with earlier (acute or subacute) stages.^10^ Also, other factors such as gradual changes in brain function near the lesion, variations in cerebral blood flow, or even changes in social circumstances and emotional well-being often develop over a longer period of time rather than within a few weeks. Importantly, recent years have seen the development of intensive neuropsychological therapy methods that lead to rapid language improvements across a short period of time (eg, 2–4 weeks) in chronic poststroke aphasia.^7–12^ Hence, comparing brain function before and after such an intensive treatment in patients with chronic stroke likely minimizes the influence of the aforementioned confounding factors. Consequently, improvements observed during such a relatively short time period can be more confidently attributed to the therapeutic intervention itself and to any cortical reorganization brought about by it, rather than to spontaneous recovery processes or other variables.

Intensive language-action therapy (ILAT), formerly called constraint-induced language therapy, stands out as an effective intensive speech and language therapy for chronic stroke PWA.^9,11–17^ Unlike approaches that focus on isolated linguistic skills, ILAT targets behaviorally relevant verbal communication within interactive social contexts.^18,19^ This method has consistently been shown to improve language function over short time periods at the chronic stage of rehabilitation. Thus, it provides a controlled environment to observe neuroplastic changes accompanying language and communication restoration. Moreover, studies have reported that ILAT also improves symptoms of depression, suggesting that language recovery and communicative interaction may contribute to better mental health.^15,17^ Therefore, we chose this method for our present study, aiming to map brain correlates of both language function and depression using functional magnetic resonance imaging (fMRI).

Traditional event-related fMRI methods rely on timing-specific stimuli, which may not be ideal for measuring blood oxygen level–dependent (BOLD) signals in patients with stroke, as stroke-related factors can attenuate and slow down BOLD responses. This can make it difficult to accurately capture therapy-related changes in brain activity.^20–23^ In contrast, measuring long-range temporal correlations (LRTC) can offer an alternative approach by evaluating the entire BOLD time series at multiple time scales. LRTC analyses quantify the temporal behavior of complex systems and have been applied across various fields in neuroscience and neuroimaging.^24–29^ In the intact brain, LRTC may indicate a proximity to the optimal neural states for the processing of information (which can be measured with BOLD fMRI). Recent research suggests that LRTC may serve as a sensitive biomarker for learning-related neuroplasticity.^30^ Specifically, decreased LRTC, which indicate a more complex and variable BOLD activity pattern over time, have been linked to greater learning gains in a motor task,^30^ as well as with increased cognitive effort^24^ or active task engagement.^31^ Recent work suggests that LRTC can also serve as a marker for depression, with studies reporting both increases and decreases in LRTC (depending on the brain regions) when comparing patients with depression to healthy controls.^32–34^ Thus, LRTC analysis not only sidesteps the challenges posed by altered hemodynamic responses in patients with stroke but also captures dynamics related to cognitive load, learning, and depression. As such, LRTC analysis represents a promising approach for assessing the neuroplastic effects of ILAT.

Many studies previously provided evidence for brain activity changes following ILAT or a similar speech and language therapy method called constraint-induced aphasia therapy or constraint-induced language therapy. For example, fMRI studies linked constraint-induced aphasia therapy–induced improvements to increased activity in perilesional areas and right hemisphere counterparts.^35,36^ MEG research from Breier et al^37^ indicated that greater pretherapy activation in left posterior language areas and their right hemisphere homologs predicted better outcomes with constraint-induced aphasia therapy, whereas Mohr et al^36^ found an increase in perilesional left-hemispheric activity across ILAT. In addition, previous EEG findings suggest that language recovery is related to increased distributed neuronal generators across both hemispheres.^38^ Building on this, this study investigated whether changes in LRTC in regions previously linked to speech and language therapy and recovery, including left perisylvian language areas and their homotopic regions in the right hemisphere, as well as domain-general areas,^39–41^ indicate neural reorganization mechanisms of language and depression improvements due to ILAT.

We hypothesize that ILAT will initiate neuroplastic changes detectable as changes in BOLD LRTC^30,31^ and that these changes will correlate with improvements in both language abilities and depression, extending the application of LRTC as a biomarker for plasticity to poststroke aphasia therapy.

## Methods

We adhered to the Strengthening the Reporting of Observational Studies in Epidemiology guidelines for reporting observational studies.^42^

### Data Availability Statement

Statistical maps generated in this study will be made available upon reasonable request by the corresponding author.

### Ethics Approval Statement

Approval for the study design was granted by the ethics review board at Charité Universitätsmedizin Berlin.

### Participants

**Figure 1.**
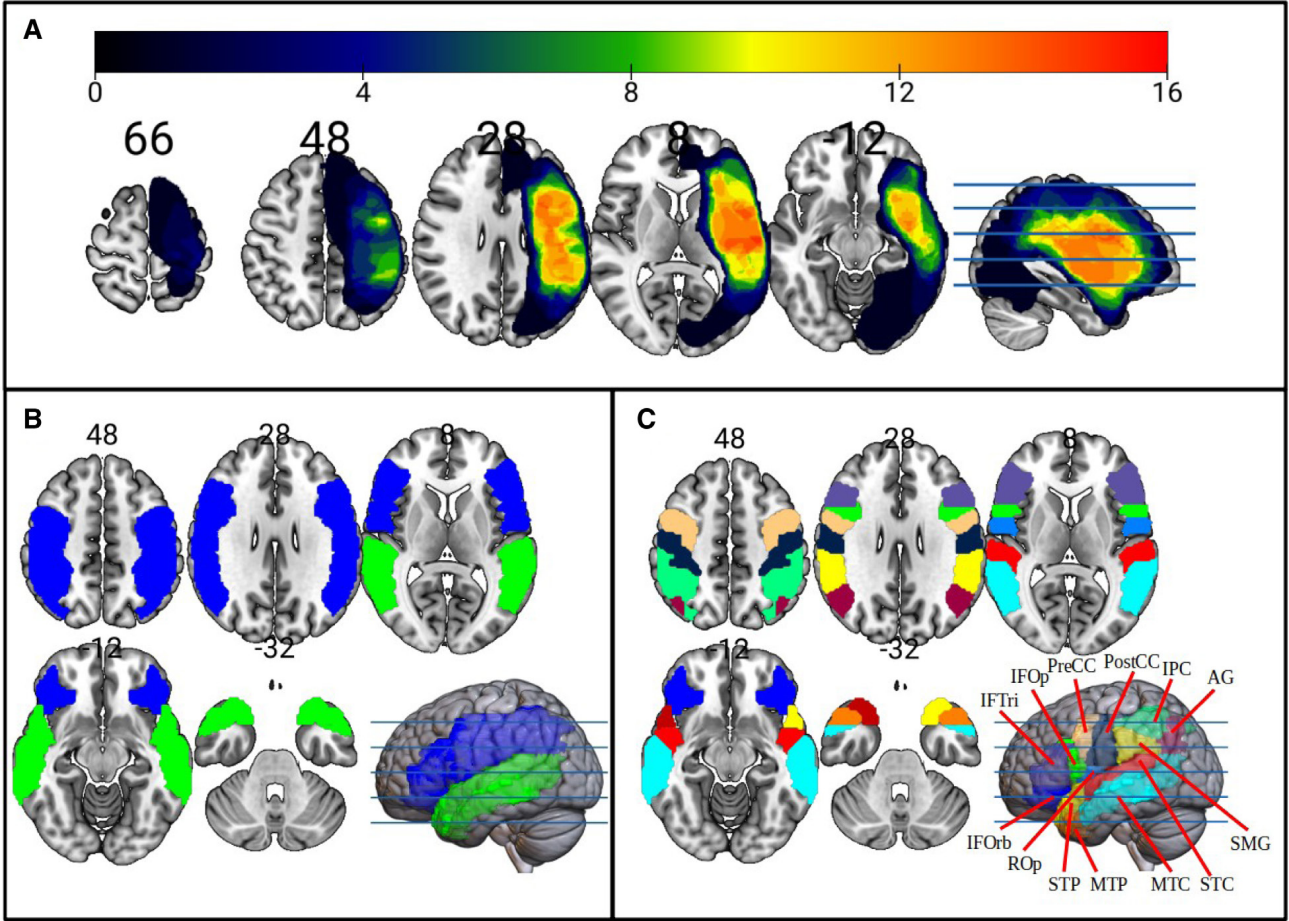
**Lesion overlay and regions of interest approaches. A**, This lesion overlay map shows the greatest lesion overlap (n=15) in the superior temporal cortex and insula. **B**, Integrated region analysis. The 4 large regions of interest, frontoparietal and temporal on the **left** and **right**, are shown on the Montreal Neurological Institute brain template. **C**, Focal perisylvian areas of interest in both hemispheres are defined using the Automated Anatomic Labeling atlas. These areas include the right and left angular gyrus (AG; magenta), inferior frontal gyrus; opercular (IFOp; green), inferior frontal gyrus; orbital (IFORb; blue), inferior frontal gyrus; triangular (IFTri; purple), inferior parietal cortex (IPC; light green), medial temporal pole (MTP; orange), middle temporal cortex (MTC; cyan), postcentral cortex (PostCC; dark blue), precentral cortex (PreCC; beige), Rolandic opercularis (Rolandic Oper; blue), supramarginal gyrus (SMG; yellow), superior temporal cortex (STC; red), and superior temporal pole (STP; black).

Right-handed participants from 3 randomized controlled trials on the efficiency of high-intensity language therapy (ILAT) for chronic poststroke aphasia were selected (for detailed inclusion criteria, see the studies by Stahl et al^12,13^). Of 59 patients, 35 did not meet fMRI safety criteria for acquiring fMRI data. Twenty-four patients underwent fMRI, 4 were excluded for incomplete sessions, and another 4 were excluded for excessive mean framewise displacement (FD; >0.5 mm). The final sample of 16 patients (9 women; mean age, 53.6 [SD, 15.7] years) largely overlaps with a previously investigated cohort.^43^ All participants were White. Additional patient data are given in Table 1, and a lesion overlay map (Figure 1A) shows overlap in the superior temporal cortex and insula. All patients consented, with approval from Charité University Hospital’s Ethics Committee, Berlin.

**Table 1.**
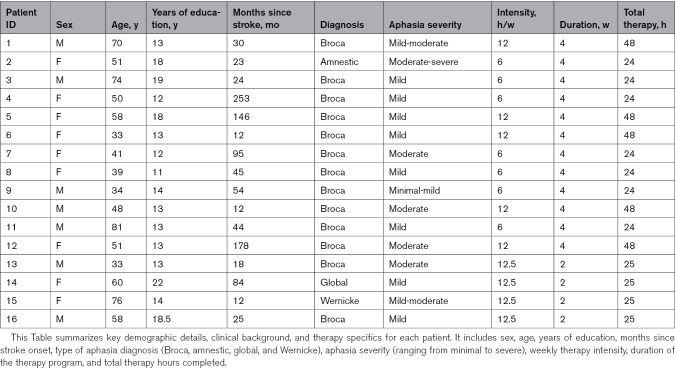
Patient Characteristics

### ILAT Therapy and Treatment Protocols

Therapy occurred from 2014 to 2020 at Freie Universität Berlin, with durations of 24 hours over 4 weeks, 25 hours over 2 weeks, or 48 hours over 4 weeks. The treatment protocol has been detailed previously.^12,13,43^ Briefly, therapy involved 2 to 3 patients and 1 therapist^18^ using pragmatic language-action games to practice language skills. Game complexity and verbal tasks were tailored to each patient’s language abilities.

### Clinical Tests

The primary outcome measure, the Aachen Aphasia Test (AAT),^44^ assessed language function and aphasia severity. We included 4 AAT subtests: the token test, repetition, naming, and comprehension. They were evaluated before (PRE) and after (POST) ILAT. Raw AAT subtest scores were converted to T scores as per AAT guidelines and then averaged to produce mean AAT (mAAT) scores for behavioral correlations. Increases in AAT T scores indicate improvements.

The Beck Depression Inventory (BDI; a self-report scale)^45^ and the Montgomery-Åsberg Depression Rating Scale (MADRS; a clinician’s/researcher’s assessment)^46^ were used to measure depressive symptoms as raw scores. To avoid expectation bias as much as possible, behavioral assessment before and after therapy was conducted by a clinical neuropsychologist not involved in delivering therapy and who was blinded to group assignment (as participants were originally part of cross-sectional and between-group study designs). The BDI and MADRS were also administered at PRE and POST. Improvements in BDI and MADRS are indicated by reductions in scores. Clinical tests, as well as fMRI (see next section), were administered 1 day before and 1 day after the therapy interval.

The AAT categorizes severity as minimal, mild, moderate, or severe based on its subtests. Overall severity was calculated by averaging these categorizations across subtests into a composite severity score, as detailed in Table 1.

### fMRI Paradigm

In the fMRI paradigm (see the studies by Dreyer et al^43,47^ for an in-depth description), patients saw single written words and strings of hashmarks; they were instructed to attend to all visual stimuli and to silently read all words. A total of 160 nouns and 120 hashmark strings were displayed for 150 ms each, in pseudorandomized order, with a randomly varying stimulus onset asynchrony between 2100 and 2500 ms.^47^ Words (mean: 7.2 characters, 2.3 syllables; frequency: 8.5/million) were sourced from the dlex corpus.^48^ Word and hashmark lengths were matched (*P*=0.89). Stimuli appeared in white uppercase on a black background and were shown in 2 8-minute runs, including nouns and hashmarks, each starting with a 15-second baseline phase featuring a fixation cross. Subsequently, functional MRI data were acquired during the reading tasks using a 3T Siemens Tim Trio scanner equipped with a 12-channel head coil and an echo planar imaging sequence (voxel size: 3×3×3 mm; TR, 2000 ms; TE, 30 ms; flip angle, 78°; 32 slices; interslice distance, 0.75 mm) followed by a T1-weighted magnetization prepared rapid gradient echo scan, conducted to obtain structural MRI data. The magnetization prepared rapid gradient echo scan parameters are given as follows: voxel size, 1-mm isotropic; TR, 1900 ms; TE=2.52 ms; TI=900 ms; flip angle, 9°; and lasted about 5 minutes.

### MRI Data Preprocessing

Lesion masks were manually drawn using MRIcroGL,^49^ covering necrotic tissue and perilesional scarring, following visual inspection and modification where necessary by 3 researchers (A.-T.P.J., A.S., and M.R.O.). POST images were aligned to PRE, and all images were normalized to the Montreal Neurological Institute with the Clinical toolbox.^50^ To account for lesion size in our main statistical analysis, we calculated the lesion volume by counting the total number of voxels within each patient’s lesion mask, following registration to the Montreal Neurological Institute.

We used Statistical Parametric Mapping 12 (7771)^51^ for fMRI data preprocessing (slice timing, realignment to the first image, coregistration, normalization, and 8-mm smoothing). Preprocessed time series from 2 8-minute sessions per timepoint was concatenated into 16-minute time series for PRE and POST. Whole-brain Hurst exponent (HE) maps were generated via detrended fluctuation analysis. FD was calculated to exclude data with a mean FD of >0.5 mm and to assess motion effects on HE maps. Correlations between mean FD and HE values were assessed to evaluate motion effects.

### Detrended Fluctuation Analysis

The detrended fluctuation analysis procedure follows the same steps as previously described in the studies by He^27^ and Jäger et al.^30^ The detrended fluctuation analysis is a technique designed to quantify the attenuation of autocorrelation within time series data, offering a measure of LRTC.^26^ The method estimates the HE, which serves as a robust measure of the degree of self-similarity in a signal over different time scales.^27^ In the context of fMRI data, detrended fluctuation analysis provides a univariate representation of the intrinsic, time-varying dynamics of individual brain regions. The HE was computed for the concatenated 16-minute fMRI data (for detailed computational steps and parameters, see Supplemental Methods S1). The estimated values provide insight into the temporal structure of fMRI signals. A higher HE suggests a slower attenuation of autocorrelations, indicating that the remote parts of the signals are correlated. On the other hand, a lower HE indicates a faster attenuation in autocorrelation and, thus, more limited temporal dependencies in the signal. Such faster attenuation corresponds to increased randomness in the data, that is, a scaling exponent of 0.5 corresponds to uncorrelated white noise.^26,52^

### Defining Regions of Interest

To investigate the contributions of bilateral perisylvian regions, 4 large regions of interest (ROIs) were generated (left frontoparietal, left temporal, right frontoparietal, and right temporal; Figure 1B). For our purposes, the perisylvian areas were defined not as the first cortical convolution surrounding the Sylvian fissure^53^ but, instead, as the set of neocortical areas defined in the Automated Anatomic Labeling (AAL)^54^ atlas, which are adjacent to this fissure. We used the AAL atlas in the Wake Forest University Pick Atlas.^55^ The frontoparietal ROIs encompassed 9 focal AAL regions, including the inferior frontal cortex (opercular, orbital, and triangular), the Rolandic opercularis, the inferior precentral/postcentral cortex (with the Montreal Neurological Institute Z<54), the angular gyrus, the supramarginal cortex, and the inferior parietal cortex. The temporal ROIs included 4 regions: the superior and middle temporal cortex, along with the superior and middle temporal pole (Figure 1B). There were 26 focal ROIs overall, 13 in each hemisphere. These focal ROIs collectively formed the larger ROIs, which will be referred to as integrated ROIs. The data from the integrated ROIs were obtained by averaging the signal across all voxels within each integrated ROI. Subsequently, an investigation of behavioral correlations was conducted in each of the 13 language-related perisylvian AAL ROIs per hemisphere, an approach that we call the focused ROI analysis (Figure 1C). To examine possible behavioral correlations in regions outside the perisylvian areas, the analysis was extended to include all 116 AAL ROIs. We acknowledge that this extensive inclusion of areas represents a low-resolution parcellation of the brain rather than a traditional ROI analysis. For clarity and consistency, we will refer to this approach as *whole-brain ROI analysis* throughout this article. All correlation analyses were conducted using Pearson r. However, because some of our behavioral variables were nonnormally distributed (see descriptive statistics in Table S1), which could compromise the assumption of a normally distributed bivariate relationship required for parametric inference with Pearson r, we obtained *P* values using a permutation test with 9999 iterations for each correlation analysis. In this test, for each pair of variables, we designated one variable as X (eg, mAAT) and the other as Y (eg, HE). We then kept the values of X constant while randomly shuffling the values of Y. This process broke any true association between the 2 variables, thereby generating a null distribution of Pearson r coefficients. The observed correlation coefficient was then compared with this null distribution, and the *P* value was computed as the proportion of permuted correlations (in absolute value) that were as extreme as the observed correlation (eg, if 500 of 9999 permuted correlations met this criterion; *P*=500/9999≈0.05). This approach provided robust significance estimates without relying on normality assumptions. The behavioral correlations in the focused and whole-brain ROI approaches were adjusted for multiple comparisons using the false discovery rate (FDR) correction method.^56^ The correction was applied separately for each analysis rather than across all analyses combined.

To improve procedure accuracy and signal-to-noise ratio, we applied individual gray matter masks to each patient’s specific ROIs and subtracted lesion masks from the left-hemispheric ROIs. HE values for each ROI at both time points (PRE and POST) were extracted using AFNI’s 3dROIstats tool.^57^

### Statistical Analyses

To assess behavioral changes pre- and post-treatment, paired samples *t* tests were conducted for mAAT, MADRS, and BDI scores. In addition, 2-tailed Pearson correlation analyses were performed to test the hypothesis that changes in language performance (mAAT) would correlate with changes in depressive symptoms (MADRS and BDI).

A 2×2×2 ANCOVA (time×hemisphere×ROI) was conducted on mean HE values in the integrated ROIs to examine time-related effects of ILAT therapy. Age and total hours of participation in ILAT, lesion volume (in voxels), and time since stroke were included as covariates.^58–60^ Interaction terms between covariates and within-subject factors (eg, time×age) were also modeled to assess moderation effects. To further explore the findings from our ANCOVA, 2 post hoc correlation analyses were conducted. The first analysis investigated the influence of age on HE changes across all integrated ROIs. For this, changes in HE from PRE to POST were calculated by averaging the signals from all integrated ROIs, and these values were then correlated (Pearson) with age to reflect the time×age interaction noted in the ANCOVA results. The second analysis aimed to determine whether age influenced behavioral improvements observed throughout the therapy. This was done by correlating age with changes in behavioral measures (mAAT, MADRS, and BDI) using permutation-based Pearson correlations.

We further investigated the relationship between HE changes and improvements in language skills and depressive symptoms by conducting permutation-based Pearson correlation analyses. These analyses related changes in HE (from PRE to POST) to changes in mAAT, BDI, and MADRS scores across the 4 integrated, 13 focused (per hemisphere), and all 116 AAL ROIs (whole-brain).

## Results

### Quality Control: Effects of Motion

No significant correlation was found between mean FD at PRE or POST and HE in any ROIs at the corresponding time points (all *P*>0.393).

### Behavioral Improvements

**Figure 2.**
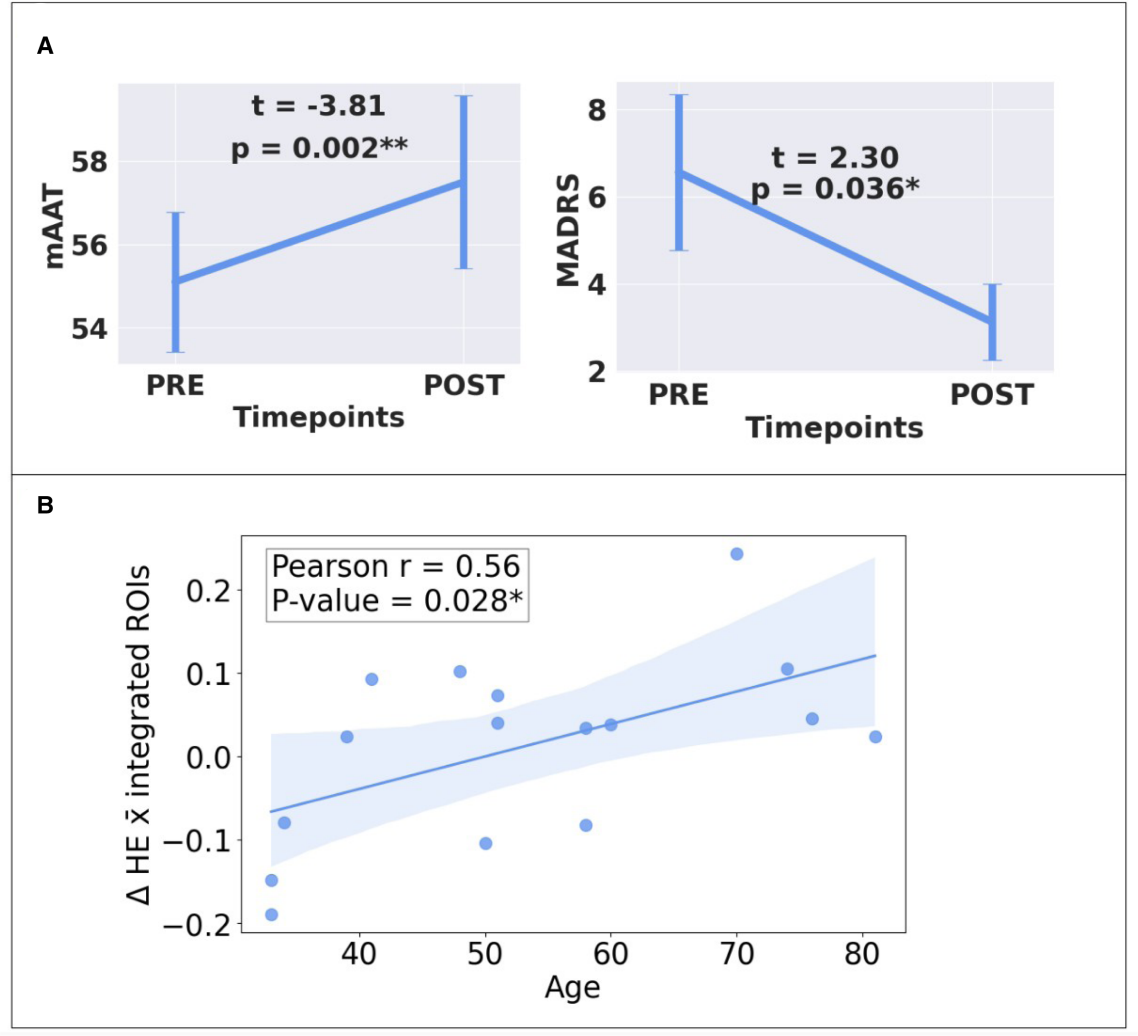
**Behavioral outcomes and age-related effects on brain signal complexity changes across therapy. A**, Changes in the mean Aachen Aphasia Test (mAAT) and the Montgomery-Åsberg Depression Rating Scale (MADRS). **B**, Post hoc correlations between age and overall Hurst exponent (HE) changes in the integrated regions of interest (ROIs). Illustrated in **A** are the results of paired samples *t* tests, indicating a significant difference between before (PRE) and after (POST) mAAT scores (**left**), and between PRE and POST MADRS scores (**right**). Also shown in **B** is the outcome of a post hoc correlation analysis between average HE change across the large integrated ROIs (as depicted in Figure 1B) and age, following a significant time and age interaction effect. The HE measures the self-similarity of the functional magnetic resonance imaging signal and serves as a putative index of learning.

Paired *t* tests showed a significant increase in mAAT scores from PRE to POST (t=−3.81; d=−0.95 [95% CI, −1.54 to −0.347]; *P*=0.002) and in MADRS scores (t=2.3; d=0.56 [95% CI, 0.036–1.10]; *P*=0.036; Figure 2A). A 2-tailed Pearson permutation correlation analysis between changes in mAAT and MADRS was performed, and the result was approaching significance (*P*=0.069). BDI also changed significantly from PRE to POST (t=3.17; d=0.79 [95% CI, 0.217–1.35]; *P*=0.006) but did not correlate with mAAT changes (*P*>0.35). However, BDI and MADRS changes were significantly correlated (r=0.60 [95% CI, 0.231–1.00]; *P*=0.008).

### Integrated ROI Analysis: LRTC Changes Over Time Across Hemispheres

The values in our HE maps typically ranged from ≈0.5 to 1, as expected based on previous fMRI literature.^26,27^

In the integrated, large-ROI analysis, the 2×2×2 (factors time [PRE/POST], hemisphere [left hemisphere/right hemisphere], and ROI [frontoparietal/temporal]) repeated measures ANCOVA with 4 covariates (see Methods section) revealed a significant time×age interaction (F=5.689; partial η²=0.341; *P*=0.036). No other interactions reached statistical significance. A post hoc permutation-based Pearson correlation analysis between overall HE change (across the integrated ROIs) and age confirmed a significant relationship (r=0.56 [95% CI, −0.162 to −0.849]; *P*=0.028; Figure 2B). Additional exploratory correlation analyses between behavioral improvements and age were not significant (mAAT: *P*>0.58; MADRS: *P*>0.435; and BDI: *P*>0.85).

### Correlations With Behavior: Language

It was tested whether changes in HE systematically relate to behavioral improvements observed between PRE and POST. A positive correlation would suggest that behavioral improvements are associated with increases in HE, indicating more stable autocorrelations in the signal over time, whereas a negative correlation would imply that improvements correspond to attenuated autocorrelations and less stability in the signal. To examine these relationships, 3 analysis approaches were used.

#### Integrated Approach

Two-tailed permutation-based Pearson correlation analyses of POST-PRE HE changes and mAAT difference scores showed significant negative correlations in 3 of 4 ROIs: right frontoparietal (r=−0.60 [95% CI, −0.844 to −0.146]; *P*=0.022), right temporal (r=−0.63 [95% CI, −0.857 to −0.192]; *P*=0.01), and left frontoparietal (r=−0.52 [95% CI, −0.809 to −0.039]; *P*_FDR_=0.039). The left temporal ROI approached significance (*P*_FDR_=0.077).

#### Focused ROI Approach

**Figure 3.**
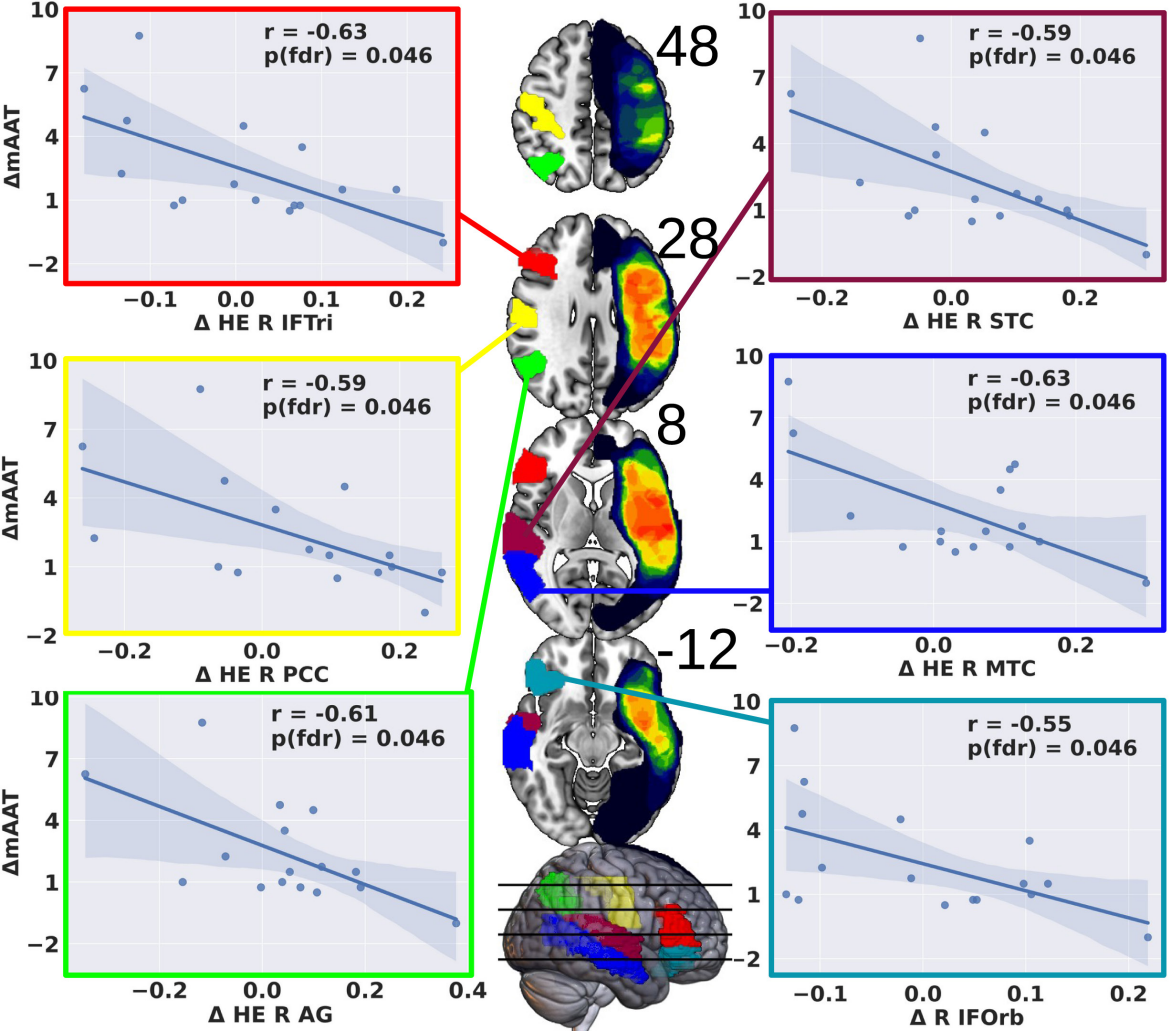
**Behavioral correlations in right hemisphere perisylvian subregions: between Δ Hurst exponent (HE) and Δ mean Aachen Aphasia Test (mAAT).** This figure illustrates the outcomes of exploratory 2-tailed correlational analyses within predefined focused perisylvian Automated Anatomic Labeling subregions of the right hemisphere. Significant, multiple comparison-corrected correlations were observed. Regions showing correlations include: inferior frontal gyrus, triangular part (IFTri, red; **top left**), postcentral cortex (PCC, yellow; **middle left**), angular gyrus (AG, green; **bottom left**), superior temporal cortex (STC, cranberry; **top right**), middle temporal cortex (MTC, blue; **middle right**), and inferior frontal gyrus, orbital part (IFOrb, light blue; **bottom right**). FDR indicates false discovery rate.

FDR-corrected 2-tailed correlations on 13 predefined AAL subregions per hemisphere yielded significant correlations in 6 right-hemispheric regions (Table 2; Figure 3).

**Table 2.**
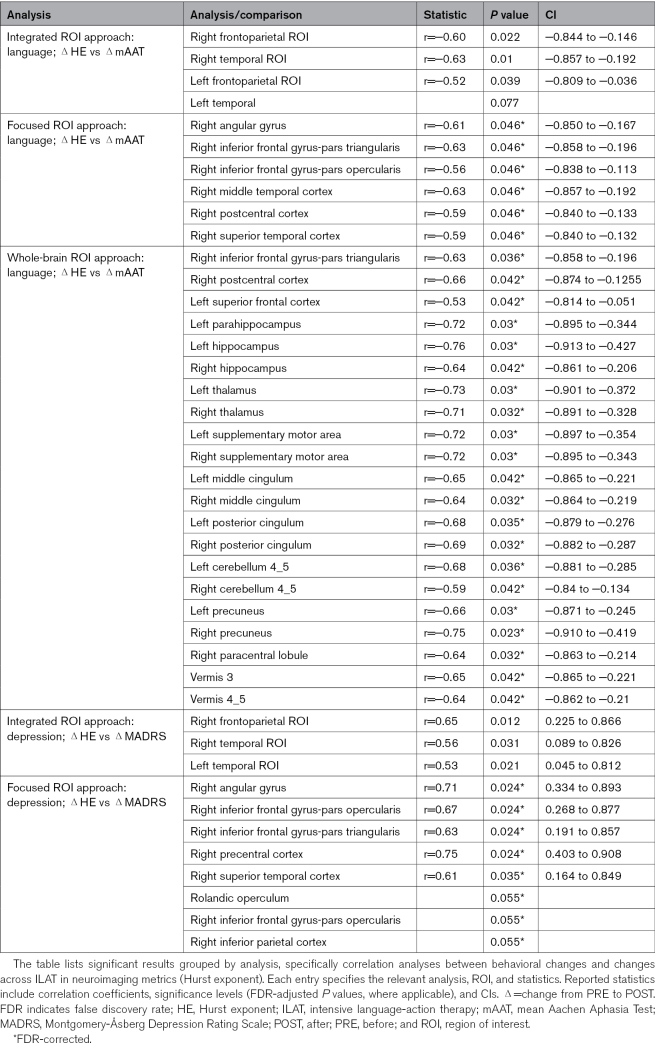
Summary of Significant Findings in the Correlation Analyses

Two-tailed analyses found no significant correlations between mAAT change and HE in left-hemispheric AAL perisylvian regions after FDR-correction (all *P*_FDR_>0.2).

#### Whole-Brain Approach

**Figure 4.**
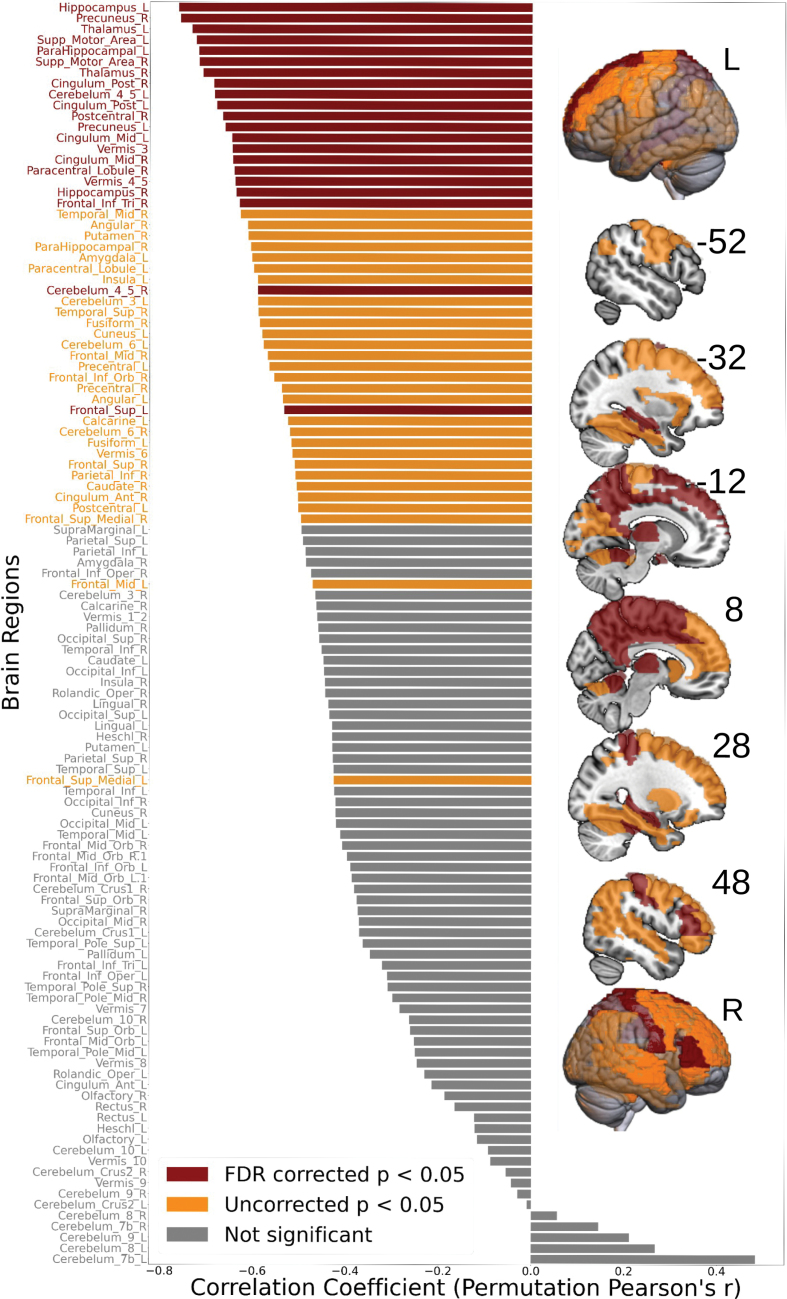
**Whole-brain region of interest (ROI) analysis: correlations with Δ Hurst exponent (HE) and Δ mean Aachen Aphasia Test (mAAT) behavioral scores.** This figure illustrates the effects of behavioral correlations across the entire brain, including all 116 Automated Anatomic Labeling (AAL) regions. Twenty-one regions, highlighted in red, showed significant permutation-based Pearson correlations that survived multiple comparison corrections using the false discovery rate (FDR). Regions in orange indicate significant correlations before correction. Nonsignificant regions are depicted in gray.

Significant FDR-corrected negative correlations between HE and mAAT changes were found in 21 of the 116 AAL areas (for a full list, see Table 2). In contrast to the focal analysis, these were mostly in multimodal areas outside the perisylvian regions (Figure 4) except for the inferior frontal gyrus, pars triangularis, and right postcentral cortex (uncropped AAL ROI). The other right perisylvian ROIs significant in the focused approach showed similar trends here (r≈0.5–0.6; uncorrected *P*<0.05) but did not survive correction across 116 regions.

### Correlations With Behavior: Depression

No significant correlations were found between BDI and HE (all *P*_FDR_>0.22). However, MADRS correlations showed significant effects:

#### Integrated Approach

Two-tailed correlations between HE changes and MADRS POST-PRE scores revealed significant positive correlations in the right frontoparietal (r=0.65 [95% CI, 0.225–0.866]; *P*=0.012), right temporal (r=0.56 [95% CI, 0.089–0.826]; *P*=0.031), and left temporal (r=0.53 [95% CI, 0.045–0.812]; *P*=0.021) ROIs.

#### Focused Approach

**Figure 5.**
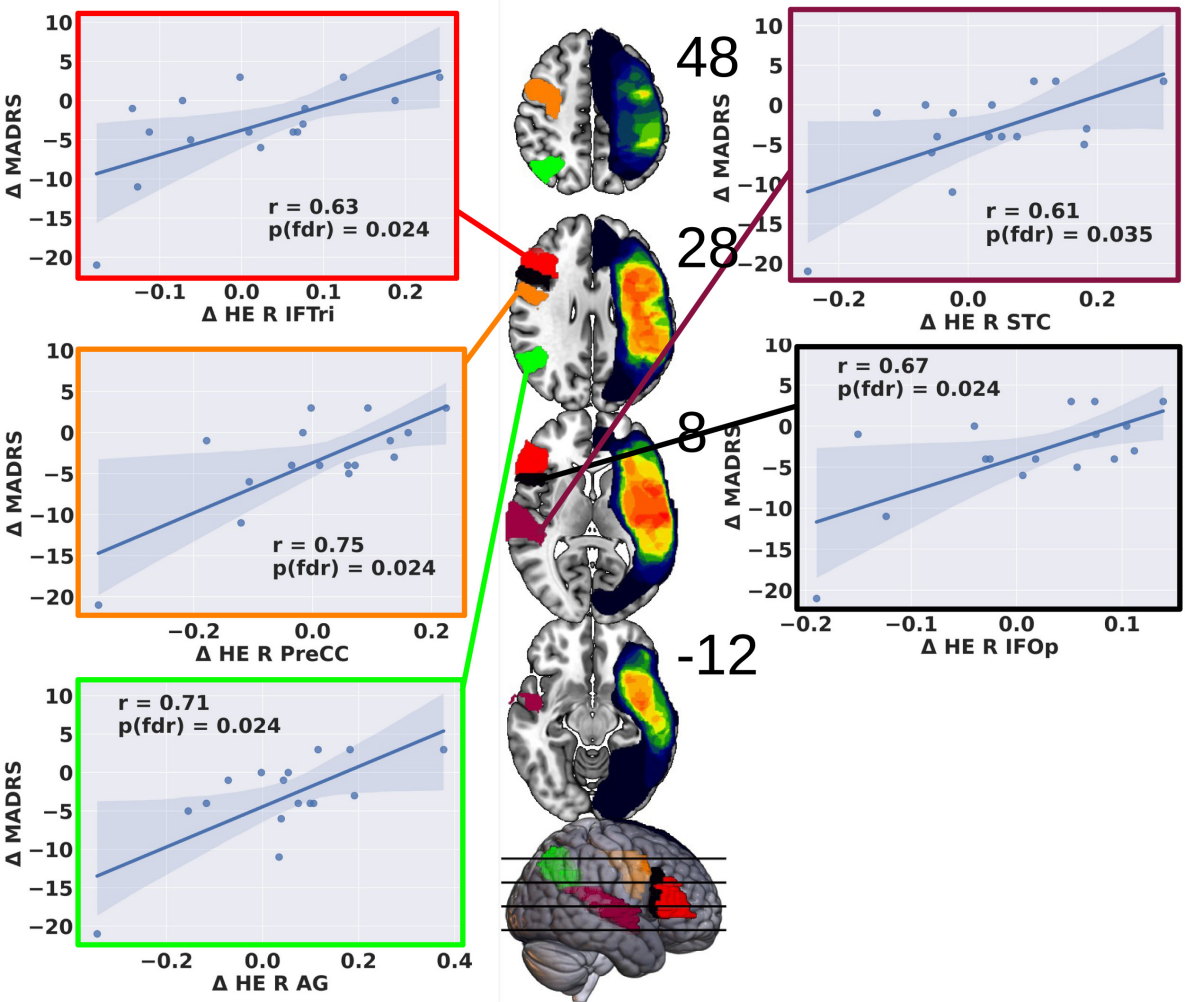
**Behavioral correlations in right perisylvian subregions: between Δ Hurst exponent (HE) and Δ Montgomery-Åsberg Depression Rating Scale (MADRS).** This figure illustrates the outcomes of exploratory 2-tailed correlational analyses within predefined focused perisylvian Automated Anatomic Labeling (AAL) subregions of the right hemisphere. Significant, multiple comparison-corrected correlations were observed. Regions showing correlations include the inferior frontal gyrus, triangular part (IFTri, red; **top left**), precentral cortex (PreCC, orange; **middle left**), angular gyrus (AG, green; **bottom left**), superior temporal cortex (STC, cranberry; **top right**), and inferior frontal gyrus, opercular part (IFOp, black; **middle right**).

Two-tailed permutation-based Pearson correlation analyses within right-hemispheric AAL subregions revealed significant positive correlations between HE changes and MADRS POST-PRE scores in 5 of 13 right perisylvian areas (Table 2; Figure 5).

#### Left Hemisphere and Whole-Brain Approaches

No significant correlations were found between HE changes and MADRS in left-hemispheric focused ROIs (all *P*_FDR_>0.086) or in the whole-brain ROI analysis (all *P*_FDR_>0.19).

All results from the correlation analyses are listed in Table 2.

## Discussion

Patients with chronic poststroke aphasia completed 2 to 4 weeks of ILAT, which involved intensive practice of language use and understanding (6–12.5 hours per week) in communicative and behaviorally relevant interactions. We acquired fMRI scans before and after therapy. Consistent with earlier reports, ILAT improved language performance^11–13^ and reduced symptoms of depression.^15^ Across therapy, younger patients, as determined by their age relative to the continuous range in our sample, exhibited greater decreases in LRTC quantified by the HE over time, whereas older patients tended toward increases. We found significant correlations between HE decreases and improvements in both language performance and depression scores. These correlations were confirmed in large bilateral regions of the frontoparietal lobes and the right temporal lobe, both above and below the Sylvian fissure. However, when focusing on focal areas defined by the AAL atlas, significant correlations survived only in right perisylvian regions, but not in the left hemisphere. Interestingly, correlations between decreases in HE and improvements in behavioral performance were similar across scales of language and depression, suggesting related improvement of language and mood as reflected by LRTC alteration. Further analysis of the AAL’s 116 areas indicated strong correlations with language performance changes in cognition-related areas, including the hippocampus, cingulate regions, supplementary motor area, thalamus, and precuneus.

### Role of Age and Bilateral Hemispheric Recruitment in Language Recovery Following ILAT

The interaction between time and age reveals a distinct pattern of LRTC across ages: younger patients (relative to the continuous age range of our sample) tend to experience larger decreases, whereas older patients exhibit more increases in LRTC (Figure 2B). Previous studies show that age affects both brain dynamics, including LRTC,^25,61–64^ and language therapy efficacy.^59,65^ For instance, healthy aging has been associated with changes in regional self-similarity,^25^ and the self-similarity of fMRI signals increases with age in older adults.^61,66^ Previous research has also suggested that aging leads to more predictable patterns in neurophysiological processes.^61^

We confirm such a pattern in our results. Age-related decline of cognitive processing and brain plasticity reduction are also well-documented,^66–68^ indicating that older individuals may face challenges in language recovery due to reduced plasticity. Behaviorally, we did not observe an age effect on either the language test or one of the depression measures although an age effect on HE was evident. While some studies have reported greater benefits from language intervention for younger than older individuals,^60,68–70^ others, including our own recent studies on ILAT, have found no consistent relationship between age and therapy success.^14,60,71–73^ Future research is needed to clarify the role of age in language therapy outcomes.

Furthermore, we found that the greatest LRTC decreases were associated with language test improvements in 3 large perisylvian ROIs (bilateral frontoparietal and right temporal) and in 6 of 13 focal perisylvian areas in the right hemisphere (inferior frontal gyrus, pars triangularis and opercularis, postcentral cortex, angular gyrus, and superior and middle temporal cortex). In light of the significant large-ROI results, the lack of significance in the focal left-hemispheric perisylvian areas may be due to a lack of statistical power; note that many of these areas were affected by the lesions, thus implicating a reduced SNR. Notably, in the whole-brain ROI analysis, the strongest correlations between behavioral and LRTC changes occurred in domain-general and memory systems, including the supplementary motor area, thalamus, precuneus, and hippocampus, all regions that have been connected to therapy-induced language reorganization.^39,41,74^ Hence, our findings provide evidence that neuroplasticity of language involves not only bilateral perisylvian areas but also domain-general as well as memory systems, which is consistent with recent reports.^36,37,39,41,75–82^ However, it should be noted that such comprehensive involvement of regions has typically been inferred from larger meta-analyses and reviews, and has rarely been observed in a relatively small cohort of 16 patients. This may indicate high sensitivity of LRTC analysis to neuroplasticity processes occurring in diverse brain areas.

### Interplay of Improvements in Depressive Symptoms and Recruitment of Perisylvian Regions in the Right Hemisphere

Post-ILAT, we observed significant decreases in depressive symptoms, confirming prior reports.^15,17^ To our knowledge, we here provide the first evidence showing that functional changes in the brain related to language improvement also mimic brain changes related to depressive symptoms. Previous research indicates that ILAT-related improvements of depressive symptoms in chronic stroke PWA may be due to sociocommunicative skills^82^ and more frequent and successful daily language use, thereby increasing social participation, self-efficacy, motivation, and mood.^15,17,82^ Our findings are consistent with such a direct link, enriching our understanding of the interplay between language rehabilitation and mental health. The data also show that intensive aphasia treatment incorporating a strong behavioral relevance, positive reinforcement, and language-action training in a group context can lead to improvements in mood. Given the compounding impact of aphasia and depression on rehabilitation,^6^ this is crucial, particularly as PWA usually do not have access to traditional psychotherapy due to their language difficulties.

Notably, LRTC changes in right hemisphere perisylvian areas correlated with changes in depressive symptoms, suggesting that right perisylvian areas are directly involved in therapy-related mood changes. However, these effects likely do not arise from changes in neural depression correlates, as whole-brain analysis showed no significant results after correcting for multiple comparisons.

While the current results provide obvious supporting evidence for a connection between language rehabilitation and depressive symptoms, there is also a more nuanced finding worth highlighting. Previous studies have demonstrated that altered BOLD LRTC are present in individuals with major depressive disorder compared with healthy controls.^34,83^ In addition, specific LRTC patterns in brain signals have been shown to reflect depressive states^33^ and social anxiety.^84^ This study is the first to link transient depressive state changes to simultaneous LRTC alterations, suggesting that LRTC changes can reflect both learning and changes in mood, possibly due to neuroplastic changes. Further investigating this area could yield valuable insights into how changes in LRTC might effectively serve as markers for changes in mood and mental health.

### Interpretation and Implications of LRTC

We observed negative correlations between LRTC changes and behavioral improvements in all 3 different ROI approaches, where decreases in the LRTC across therapy were related to greater gains in language performance, whereas LRTC increases were associated with small or absent gains.

Reduced LRTC indicates more randomness in the brain’s BOLD signal with attenuated autocorrelations over time as a result of language therapy. Increased randomness in fMRI signals, considered a marker of cognitive effort,^24^ neuroplasticity, and learning,^30^ as well as cognition in general,^31^ may reflect the brain’s ability to process information more effectively. Increased randomness in the neural system can alter neural responsiveness by increasing the variability of neuronal firing.^85^ Hence, as hypothesized before,^30^ decreased LRTC may indicate ongoing plasticity in brain regions processing new information. Such theories imply that lower LRTC, indicating increased temporal randomness of the BOLD signal resulting from therapy, may signify an active learning state where the brain is optimizing its information processing capabilities.

Using LRTC as a biomarker for learning-related neuroplasticity offers practical benefits in neuroplasticity research following interventions. It eliminates the need for active patient involvement during MRI scans, which is particularly advantageous for patients with stroke who might struggle with timing-specific tasks due to comorbidities or small vessel disease that could delay the BOLD response.^86^

As noted above, our findings suggest that LRTC may offer greater sensitivity to learning-related neuroplasticity than conventional univariate methods, as evidenced by the identification of widespread regional involvement previously observed primarily in meta-analyses.^39–41^ Hence, LRTC may more sensitively capture learning-related neuronal dynamics. Examining it in parallel with cognitive and emotional recovery could offer a more nuanced understanding of neuroplasticity during language rehabilitation.

### Limitations

Generalizability of the present findings is limited by the small sample size (N=16) and by including only patients able to tolerate and complete fMRI. In addition, the small sample size may have contributed to a lack of significant findings in our whole-brain analysis of depression measures. Motor speech disorders (eg, apraxia of speech) were not assessed as they were beyond the scope of this study. It would be interesting to investigate in future research whether additional motor speech disorders have an impact on language recovery and its neural correlates.

In addition, the person conducting the behavioral assessments could not be blinded to the timepoint, as each patient was assessed twice by the same tester. This feature could represent a potential source of expectation bias and should be considered when interpreting the observed behavioral improvements. Further research is needed to clarify the impact of ILAT on depression and language.

### Conclusions

We observed LRTC reductions across short-term ILAT in relatively young patients with chronic poststroke aphasia, whereas older patients showed an increase. ILAT led to significant improvements in both language function and mood. In addition, LRTC changes in perisylvian areas were associated with changes in language performance, particularly in the right hemisphere, as well as in domain-general and memory regions, including the supplementary motor area, thalamus, precuneus, and hippocampus. LRTC changes in the right perisylvian areas also correlated with changes in mood, suggesting a possible link between language and mood recovery. Greater LRTC decreases emerged as markers of more pronounced learning gains, as previously hypothesized, particularly in younger patients. These results show that ILAT leads to cognitive and affective recovery in chronic poststroke aphasia and suggest that LRTC could be a sensitive biomarker for therapy-induced neuroplasticity in clinical settings.

## Article Information

### Acknowledgments

The authors thank Verena Arndt for her support at different stages of this work. A text-generating artificial intelligence tool was used to enhance the readability and clarity of this article.

### Sources of Funding

This work was supported by the Deutsche Forschungsgemeinschaft grants PU-97/15-1 & 2 to Dr Pulvermüller and 697/5-2 to Dr Mohr, the European Research Council (ERC) grant ERC-ADG 883811 to Dr Pulvermüller, the Heart and Stroke Foundation of Canada New Investigator Award and Catalyst from the Canadian Institutes of Health Research (grant HNC 170723) to Dr Steele, and the Fonds de Recherche Santé Chercheurs-boursier (grant FRQS CB Junior 2 349443) to Dr Steele.

### Disclosures

None.

### Supplemental Material

Supplemental Methods S1

Table S1

STROBE Checklist

## Supplementary Material


