# Supplementary material for: BOLD Long-Range Temporal Correlations Reflect Changes in Language and Depression Across Intensive Aphasia Therapy
Source: Stroke. 2025 Sep 10;56(11):3138–52. doi: 10.1161/STROKEAHA.124.050064 (PMC12551423; doi:10.1161/STROKEAHA.124.050064)
Supplement: Supplementary file 1 [file str-56-3138-s001.pdf]

Supplemental Material

Supplemental Methods S1. Computational Steps of DFA:

1. Cumulative Summation and Mean Removal:

Given a timeseries x(t) of length N, the cumulative sum is computed after subtracting the mean:

$$X(i)=\sum_{t=1}^{i}\left(x(t)-\bar{x}\right)$$

where x¯ is the mean of x(t).

2. Segmentation into Logarithmically Spaced Windows:

The cumulative signal X(i) is divided into logarithmically spaced windows of size s, ensuring a consistent scaling of fluctuation calculations. In this study, five windows were used, ranging from 16 to 52 samples (window sizes: 16, 21, 27, 36, 52).

3. Local Detrending (Linear Regression):

Within each window s, a least-squares linear regression is applied to remove local trends (captured with X*s*). The root-mean-square fluctuation of the detrended fluctuations is then computed:

$$F(s)=\sqrt{\frac{1}{N}\sum_{i=1}^{N}\left(X(i)-X_s(i)\right)^2}$$

and then averaged across all windows of the same size

4. Scaling Relationship and HE Estimation:

The fluctuation function F(s) is plotted against the window size s on a log-log scale. The Hurst exponent (HE) is estimated as the slope of a least-squares fit:

$$logF(s)=Hlogs+C$$

5. Interpretation of HE Values: H=0.5 corresponds to a random, uncorrelated process (white noise). 0.5<H<1 indicates persistent long-range dependencies, where larger values suggest stronger LRTC.

Table S1. Descriptive statistics for neuroimaging, demographic, and behavioral variables across, pre- and post-therapy. This table provides the mean, standard deviation (SD), median, minimum (Min), maximum (Max), skewness, kurtosis, and Shapiro–Wilk test values for each variable relevant to the analyses. R = Right; L = Left; Δ = Change from pre- to post-therapy; Lesion volume is reported in voxels; Time since stroke in months; Age and Years of education in years; mAAT = Mean scores in the Aachen Aphasia Test; BDI = Beck's Depression Inventory; MADRS = Montgomery–Åsberg Depression Rating Scale.

Variable	Mean	SD	Median	Min	Max	Skewness	Kurtosis	Shapiro-Wilk (W)	Shapiro-Wilk (p)
R frontal PRE	0.78	0.12	0.78	0.6	1.03	0.36	-0.79	0.966	0.763
R frontal POST	0.81	0.12	0.8	0.63	1.01	0.29	-0.89	0.954	0.558
Δ R frontal	0.02	0.12	0.05	-0.23	0.22	-0.53	-0.22	0.968	0.799
L frontal PRE	0.74	0.13	0.78	0.47	0.98	-0.38	-0.38	0.973	0.878
L frontal POST	0.76	0.15	0.76	0.5	0.93	-0.35	-1.22	0.899	0.078
Δ L frontal HE	0.01	0.12	0	-0.22	0.23	-0.05	-0.55	0.982	0.977
R temporal PRE	0.89	0.12	0.86	0.72	1.12	0.38	-0.99	0.944	0.405
R temporal POST	0.91	0.13	0.88	0.73	1.16	0.53	-0.58	0.948	0.452
Δ R temporal	0.02	0.12	0.03	-0.23	0.28	-0.14	0.25	0.96	0.665
L temporal PRE	0.84	0.15	0.85	0.51	1.08	-0.32	-0.09	0.976	0.924
L temporal POST	0.84	0.14	0.86	0.59	1.11	-0.06	-0.71	0.984	0.986
Δ L temporal	0	0.13	0	-0.19	0.23	0.16	-1.1	0.957	0.606
Age	53.56	15.66	51	33	81	0.27	-1.06	0.938	0.32
Time Since Stroke	65.94	70.14	37	12	253	1.52	1.31	0.771	0.001
Lesion Volume	165344.44	87462.24	144996	36182	304815	0.15	-1.38	0.93	0.245
mAAT PRE	55.09	6.75	57.5	42.75	65.75	-0.36	-1	0.941	0.365
mAAT POST	57.5	8.33	58.38	43.25	70.75	-0.13	-1.19	0.953	0.542
Δ mAAT	2.41	2.52	1.5	-1	8.75	1.15	0.62	0.871	0.028
BDI PRE	20.44	14.96	23.5	0	51	0.22	-0.77	0.949	0.474
BDI POST	11.06	10.79	8.5	0	39	1.33	1.13	0.856	0.017
Δ BDI	-9.38	11.84	-8	-26	10	0	-1.39	0.919	0.164
MADRS PRE	6.56	7.14	5	0	27	1.57	2.22	0.822	0.005
MADRS POST	3.12	3.5	2	0	11	0.95	-0.22	0.849	0.013
Δ MADRS	-3.44	5.98	-3.5	-21	3	-1.6	2.79	0.827	0.006
